# Metabolic reprogramming heterogeneity in chronic kidney disease

**DOI:** 10.1002/2211-5463.13568

**Published:** 2023-02-14

**Authors:** Verónica Miguel, Rafael Kramann

**Affiliations:** ^1^ Institute of Experimental Medicine and Systems Biology RWTH Aachen University Hospital Aachen Germany

**Keywords:** fatty acid oxidation, glycolysis, kidney fibrosis, mitochondria, myofibroblasts, tubular epithelial cells

## Abstract

Fibrosis driven by excessive accumulation of extracellular matrix (ECM) is the hallmark of chronic kidney disease (CKD). Myofibroblasts, which are the cells responsible for ECM production, are activated by cross talk with injured proximal tubule and immune cells. Emerging evidence suggests that alterations in metabolism are not only a feature of but also play an influential role in the pathogenesis of renal fibrosis. The application of omics technologies to cell‐tracing animal models and follow‐up functional data suggest that cell‐type‐specific metabolic shifts have particular roles in the fibrogenic response. In this review, we cover the main metabolic reprogramming outcomes in renal fibrosis and provide a future perspective on the field of renal fibrometabolism.

## Graphical abstract

Chronic kidney injury drives cell‐type‐specific metabolic alterations with particular roles in fibrogenesis. Proximal tubular epithelial cells suffer mitochondrial damage and fatty acid oxidation impairment, triggering different cell death programs and cytokine release, attracting/activating immune cells and fibroblasts. Macrophage polarization also involves metabolic adjustments. Activated fibroblasts (myofibroblasts) are reprogrammed toward increased aerobic metabolism to acquire an extracellular matrix synthetic phenotype.

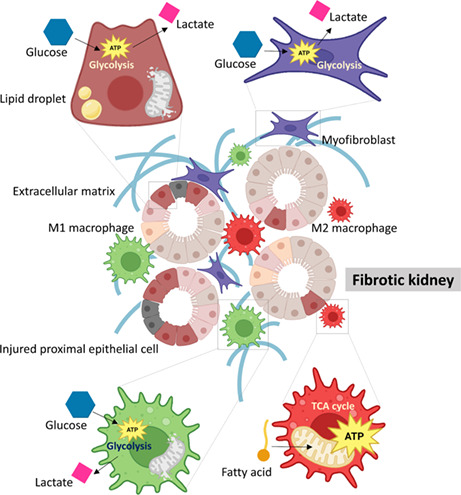

**Abbreviations**
CKD: chronic kidney disease
ECM: extracellular matrix of proteins
ESRD: end‐ stage renal disease
FA: fatty acid
FAO: fatty acid oxidation
FR‐PTC: failed repair proximal tubule cell
IL: interleukin
MPTP: mitochondrial permeability transition pore
OXPHOS: oxidative phosphorylation
PDGF: platelet derived growth factor
PT‐AcInj: acute injury proximal tubule cell
PTEC: proximal tubular epithelial cell
PT‐R: repairing proximal tubule cell
ROS: reactive oxygen species
TCA: tricarboxylic acid
TEC: tubular epithelial cell
TGF: transforming growth factor
TNF: tumor necrosis factor

## Chronic kidney disease

Chronic kidney disease (CKD) is a clinical condition that involves the progressive deterioration of renal function and represents an important medical, social, and economic burden with high morbidity and mortality rates. It affects about 10% of the worldwide population and is present in around 30–40% of patients with highly prevalent pathologies, such as hypertension and diabetes mellitus. CKD is predicted to be the 5^th^ most important cause of death worldwide within the next 20 years [1]. Current CKD therapies are limited to immunosuppression in some fast‐progressive diseases or systemic blood pressure control. Although recent trials have reported promising results with SGLT2 inhibition, the majority of patients with CKD still progress toward end‐stage renal disease, which requires lifelong dialysis or kidney transplantation, creating an enormous unmet clinical need [2].

Regardless of etiology, progression of CKD includes the development of tubulointerstitial and glomerular fibrosis as a consequence of the excessive deposition of extracellular matrix proteins (ECM) along with persistent inflammation, tubular epithelial cell (TEC) dedifferentiation, and rarefaction of the peritubular microvasculature [3]. Myofibroblasts, derived from fibroblasts and PDGFRβ^+^/PDGFRα^+^ mesenchymal cells, are the principal ECM‐producing cells in the kidney [4]. Injury of the tubular epithelium occurs across most types of kidney disease at different stages and can, for instance, be caused by hypoxia, toxic compounds, or proteinuria with subsequent dedifferentiation of epithelial cells or even cell death. Sublethal damaged injured TECs may be drivers of fibrogenesis by releasing paracrine proinflammatory or profibrotic mediators [5]. Transforming growth factor‐β (TGF‐β) is the principal profibrogenic cytokine [6]. In addition, a variety of cytokines, such as interleukin 1 (IL‐1) and tumor necrosis factor (TNF), other key fibrogenic factors including platelet derived growth factor (PDGF), connective tissue growth factor, fibroblast growth factor 2, TNF‐like weak inducer of apoptosis (TWEAK) and Ang II [7], which involve the activation of TGF‐β, Notch, Wnt/β‐Catenin, PDGF, and Ang II/Reactive oxygen species (ROS)‐signaling pathways, are all considered crucial for kidney fibrosis development [8, 9, 10, 11, 12].

**Fig. 1:**
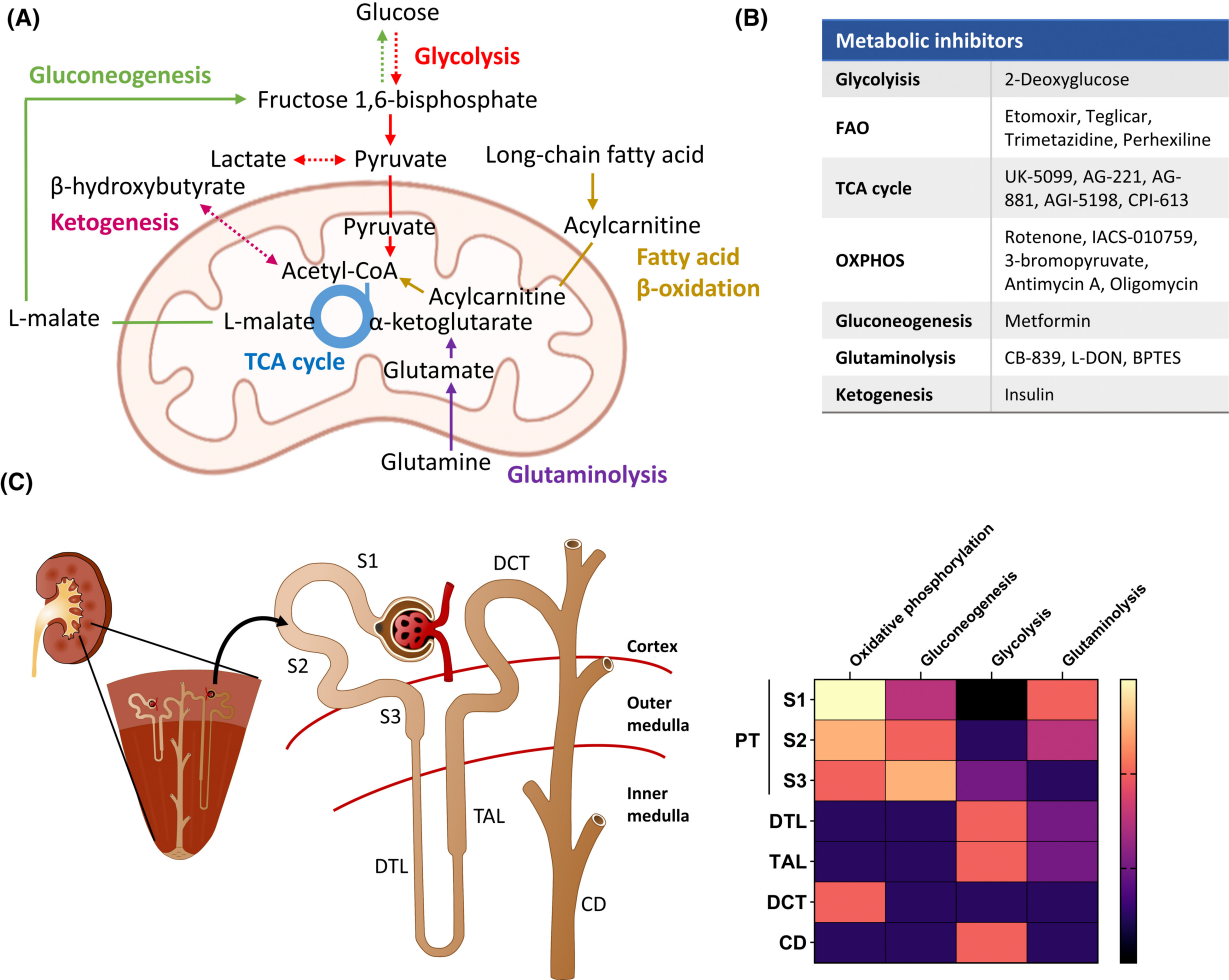
Renal metabolism within nephron tubular segments. (A) Major metabolic pathways in the kidney. Arrows with the same color link compounds from the same metabolic pathway. Forward arrows represent direct one‐step reactions, dashed arrows represent multistep reactions, and equilibrium arrows represent reversible reactions. (B) Examples of drugs targeting metabolic pathways. (C) Major metabolic pathways in each nephron tubular segment. The heat map indicates the approximate relative activation level of metabolic pathways within the tubular cells from each nephron segment. The scale bar ranges from yellow to black (high to low activation level). CD, collecting duct; DCT, distal convoluted tubule; DTL, descending limb of loop of Henle (LoH); S1, Segment 1; S2, Segment 2; S3, Segment 3; TAL, thick ascending limb of LoH.

Experimental and clinical evidence obtained in the last few years have fostered a reinterpretation of the critical role of alterations in the main metabolic programs in the pathophysiology of renal fibrogenesis (Fig. 1A) [13]. They suggest that a cell‐type‐specific metabolic shift underlies the fibrogenic phenotype, whose understanding results essential to explore metabolism‐based effective therapeutic alternatives.

## Metabolic alterations in renal fibrogenesis

**Fig. 2:**
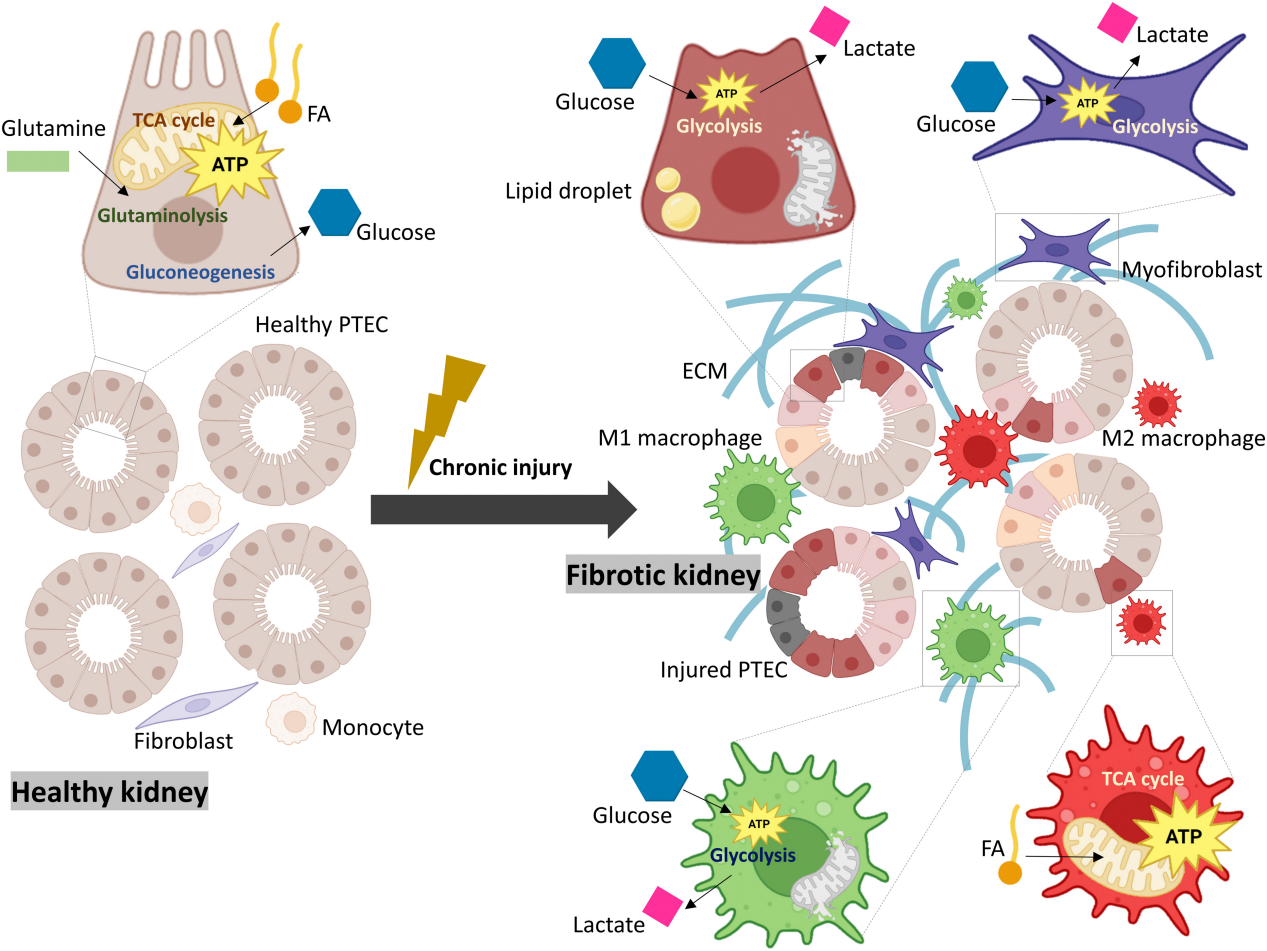
Cell‐type metabolic alterations in renal fibrogenesis. In CKD, PTECs undergo mitochondrial damage and impairment of FAO (their main energy source), triggering different cell death programs and the release of proinflammatory and profibrotic cytokines, attracting and activating immune cells and fibroblasts. M1 proinflammatory macrophage polarization mainly involves enhanced glycolysis and the blockage of the TCA cycle, while M2 macrophages, with important roles in wound healing and resolving inflammation, are more dependent on OXPHOS. Myofibroblasts are reprogrammed toward increased aerobic metabolism to acquire an ECM synthetic phenotype.

Infiltration of inflammatory cells, including lymphocytes, macrophages, dendritic cells, and mast cells, is a key feature of early stages of tubulointerstitial fibrosis [14]. The relationship between metabolism and immune cell function has gained momentum in recent years. Dramatic switches in cellular metabolism involve phenotypic and functional changes in macrophages. They are classically divided into two types: M1 and M2 according to their activation state and functions. The M1 proinflammatory polarization takes place during the early phase of renal injury and can be triggered by several stimuli including interferon gamma (IFN‐γ) and lipopolysaccharide *in vitro*. Macrophages acquire an M2 phenotype throughout the later stages of tissue repair in response to IL‐4, IL‐13, and IL‐1β and play important roles in wound healing and resolving inflammation. M1 macrophages are more dependent on glycolysis and present two breaks in the tricarboxylic acid (TCA) cycle, while M2 cells mainly rely on oxidative phosphorylation (OXPHOS; Fig. 2) [15]. Although a protective role for anti‐inflammatory M2 macrophages has been increasingly reported, their phenotypic plasticity and the conflicting macrophage phenotypes *in vivo* are major barriers for their therapeutic application [16].

Myofibroblasts also seem to undergo metabolic reprogramming characterized by increased aerobic glycolysis to meet the fast energy demand imposed by the ECM synthetic phenotype (Fig. 2) [17]. Beyond providing a rapid energy‐generating mechanism compared with OXPHOS, glycolysis produces by‐products such as lactate, which can affect other profibrotic pathways, such as TGF‐β signaling. It has been reported that chicken ovalbumin upstream promoter‐transcription factor II (COUP‐TFII), a transcription factor which directly regulates PGC‐1α expression, triggers a metabolic shift in human and mouse myofibroblast metabolism toward enhanced glycolysis and the generation of profibrotic mediators [18]. Inhibition of aerobic glycolysis has emerged as a potential antifibrotic strategy by reducing myofibroblast activation [19, 20, 21]. However, we still lack a complete understanding of stromal cellular heterogeneity. Different stromal subsets likely also differ in their metabolism in homeostasis and disease [22, 23]. In this review, we will focus on the region‐specific metabolic perturbations of proximal renal tubular cells, the better metabolically characterized cell type in renal fibrogenesis.

### Proximal renal tubular cells

The kidney is the second organ in terms of mitochondrial content and oxygen consumption after the heart [24]. This is partially due to proximal tubules, which constitute ~ 50% of the total kidney cell number and require a high degree of active transport as they reabsorb 80% of the filtrate that traverses the glomerulus. Mitochondrial metabolism and particularly, fatty acid oxidation (FAO), is the preferred energy source, likely as an adaptation to generate large amounts of adenosine triphosphate (ATP), which also determines proximal tubular epithelial cell (PTEC) fate and function [25]. PTECs also rely on gluconeogenesis. Kidney is the only organ, aside from the liver, able to perform this metabolic program, contributing up to 20% of all glucose production (Fig. 1C) [26]. By contrast, podocytes, endothelial cells, and mesangial cells exhibit a lower degree of oxidative capacity also likely adapted to their function since their role requires less energy [27]. Therefore, distinct segments within the mature nephron utilize metabolic pathways likely adapted to their specific function. This feature is also conserved in the human nephron and has allowed to infer the susceptibility of each tubular segment toward ischemia [28]. Missing capability for anaerobic energy generation combined with its basal low pO_2_ suggests S3 segment of the proximal tubule as a primary site for hypoxia‐based injury [29].

#### FAO and oxidative phosphorylation

Recent single‐cell RNA sequencing (scRNA‐seq) studies suggest considerable heterogeneity in PTEC states during fibrosis, including acute injury (PT‐AcInj), repairing (PT‐R), and failed repair proximal tubule cells (FR‐PTC), in addition to healthy S1/S2/S3 PTEC [30, 31]. Although FAO is transiently activated in PTECs (marked by Perilipin 2 (PLIN2) expression, a marker of intracellular lipid droplets) at the very early stages of ischemia–reperfusion injury and is associated with successful repair [32], its impairment has gained recognition as a potential pathogenic factor in the progression of kidney disease (Fig. 2). Defective amino acid transport and catalysis is also present in FR‐PTCs and is associated with CKD progression [33, 34]. Branched‐chain amino acids may provide TCA cycle intermediates in the absence of FAO [35]. Patients with CKD exhibit a drastic reduction of FAO and OXPHOS coupled with intracellular lipid deposition in the proximal tubular compartment, along with increased accumulation of short‐ and middle‐chain acyl‐carnitines, reflecting impaired FAO [36, 37, 38]. Several transcription factors, such as peroxisome proliferator‐activated receptor (PPAR)‐α, an important regulator of FAO genes, mitochondrial transcription factor A (TFAM), essential for mitochondrial encoded gene transcription and replication, as well as estrogen‐related receptor α (ESRRα) and PPARγ coactivator 1α (PGC1α), which regulate mitochondrial biogenesis, show decreased expression in patient samples and mouse models with CKD [39, 40]. Mechanistically, TGF‐β‐driven SMAD3 binding overlaps with the active enhancer histone tail modification H3K4me1 of the Ppargc1a promoter sequence, regulating the levels of proteins involved in fatty acid (FA) uptake and oxidation [39, 41]. Piret *et al*. [42] have proposed a novel transcriptional regulatory mechanism, involving a decrease in the expression of Krüppel‐like factor 15 (KLF15) in FAO loss. They have identified a close proximity in the binding sites of KLF15 and PPAR‐α in the promoters of the FAO genes Carnitine palmitoyl‐transferase 1 A (Cpt1a) and Acetyl‐CoA acyltransferase 2 (Acaa2). Post‐transcriptional targeting of Cpt1a by miR‐33, miR‐150, and miR‐495 has also been reported in human PTECs under a profibrotic insult *in vitro* [43, 44].

A prevailing line of evidence is that defects in energy production exert a negative impact beyond mere lipotoxicity. Although the inhibition of CD36‐, FATP2‐, or KIM‐1‐mediated FA uptake ameliorated experimental tubulointerstitial inflammation and fibrosis, enhanced lipid overload by tubular CD36 overexpression did not trigger spontaneous renal fibrogenesis in mice [39, 45, 46]. CPT1A is a FA mitochondrial shuttling enzyme, which catalyzes the FAO rate‐limiting step [47]. Both genetic deletion of Cpt1a and its pharmacological inhibition with etomoxir uncoupled mitochondrial function, aggravating tubular injury and interstitial fibrosis [39, 48]. Conversely, augmentation of FAO by CPT1A overexpression protected the tubule from injury [37]. The protective role of FAO restoration is also supported by indirect lines of evidence. Increased tubular expression of PGC‐1α and TFAM, and treatment with fenofibrate (a PPAR‐α agonist), C75 (a FA synthase blocker and CPT1A activator) or AICAR (an AMPK activator, which induces Cpt1a expression and reduces the levels of its physiological inhibitor, malonyl‐CoA) reduced experimental kidney fibrosis and associated functional impairment [39, 49, 50, 51]. The future development of specific, nontoxic CPT1 activators may pave the way to confirm this prospect.

Under energy failure, ketones can be important substrates for the kidney. Lipolysis and FAO result in high levels of acetyl‐Co A that, when the oxidation capacity of the TCA cycle is exceeded, is instead diverted toward acetoacetate, which is subsequently reduced to β‐hydroxybutyrate. This process is called ketogenesis and occurs almost exclusively in liver mitochondria, but ketones can be reabsorbed by PTECs and be used for mitochondrial energy production and signaling purposes [52]. Physiological levels of ketones exert renoprotective effects based on their anti‐inflammatory and antioxidant properties. In fact, ketones are proposed to mediate the benefits of SGLT2 inhibitors in the kidney [53].

Alterations in mitochondrial dynamics and quality control also affect CKD development due to the cells´ failure to meet their energetic needs. Enhanced fragmentation of mitochondria has been observed in kidney tubules from CKD patients and mouse models. PTEC deletion of dynamin‐related protein (DRP1), the main regulator of mitochondrial fission, or its pharmacological inhibition with the drug Mdivi‐1, preserved mitochondrial structure and protected against renal fibrosis [54]. Conversely, PTEC deficiency of Mitofusin 2 (MFN2), which controls outer mitochondrial membrane fusion, accelerated kidney function recovery and enhanced survival after renal ischemia in mice [55]. Under the same type of injury, absence of OMA1 reduced the proteolysis of OPA1, which is involved in inner mitochondrial membrane fusion, ameliorating renal tubular damage [56]. Mitophagy selectively degrades damaged mitochondria via the autophagy machinery, which is triggered by the loss of mitochondrial membrane potential. Knockout mice for PINK1 or PARKIN genes, involved in mitophagosome formation, developed severe mitochondrial damage and kidney tubule cell death [57]. Conversely, enhancement of mitophagy has emerged as a strategy to improve mitochondrial health and provide protection against renal fibrosis. Thus, Bcl2‐interacting protein (BNIP)3 or BNIP3L (NIX), PINK1/PARKIN‐independent mitophagy receptors, ameliorate kidney damage [58, 59].

Damaged mitochondria release ROS and can lead to the cytosolic leakage of mitochondrial DNA (mtDNA), which activates cytosolic nucleotide sensors and induces inflammation. Pharmacological inhibition of the cytosolic DNA‐sensing cGAS–STING signaling pathway using C176, or genetic deletion of STING attenuates kidney tubule injury and fibrosis [51]. Knockout mice for AIM2, involved in another cytosolic nucleotide sensing pathway, showed reduced caspase‐1 activation and were protected from renal fibrosis and pyroptosis, a proinflammatory programmed cell death [60, 61]. Mitochondrial injury also triggers other cell death mechanisms including apoptosis, necroptosis, and ferroptosis, releasing proinflammatory cytokines which contribute to immune cell attraction or fibroblast activation. Apoptosis is a type of cell death, which occurs without the rupture of the cell membrane. The release of cytochrome c from the mitochondria through BAX/BAK pores promotes the oligomerization of a cytochrome c/Apaf‐1/caspase‐9 complex (apoptosome). It results in the activation of the initiator caspase‐9, which cleaves and activates downstream effector caspases‐3 and ‐7, leading to widespread substrate cleavage [62]. Attenuation of renal tubular cell apoptosis in BAX/BAK double KO mice prevented renal failure after ischemia [63]. Tumor necrosis factor‐alpha promotes necroptosis (programmed necrosis) by receptor‐interacting protein kinase‐3 (RIPK3)‐mediated NADPH oxidase‐4 (NOX4) recruitment to the mitochondria or through the mitochondrial permeability transition pore [64]. Ferroptosis is a caspase‐independent form of cell death characterized by iron‐dependent oxidative stress‐induced lipid peroxidation, leading to plasma membrane rupture, which underlies a TEC maladaptive repair [65, 66]. However, further research is needed to understand the relationship between different types of cell death and to address whether they determine an adaptive or maladaptive kidney repair response.

#### Glucose and glutamine metabolism

Proximal tubular epithelial cells also rely on gluconeogenesis to maintain glucose homeostasis by generating glucose from noncarbohydrate molecules. In the kidney, lactate is the principal substrate (50%) of gluconeogenesis, followed by glutamine (20%) and glycerol (10%). PTEC gluconeogenic function is impaired in CKD human biopsies and murine models and is associated with a worse renal prognosis (Fig. 2) [67]. Although PTECs produce and reabsorb glucose (mostly via SGLT2 on the apical membrane of S1‐2 segments and to a lesser extent via SGLT1 in the S3 segment), it is released into the circulation. With the exception of S3 segment, PTECs are almost unable to use glucose as energetic substrate. This is likely due to the closer localization of the S3 segments to the outer medulla and hence, to the limitation of O_2_ levels, compared with the S1 and S2 ones, which are restricted to the renal cortex. TECs in the more distal segments of the nephron exhibit enhanced glycolysis and progressively lose their capacity to carry out gluconeogenesis and OXPHOS (Fig. 1C). Under ischemic injury, PTECs are prompted to perform glycolysis instead of FAO, as reflected the accumulation of glycolysis intermediates and the upregulation of glycolytic enzymes [68]. Consistently, more lactate is present in FR‐PTs [69]. This is even maintained at late stages postreperfusion and in obstructive mouse models. Consistently, kidney metabolomics and RNA sequencing data from CKD patients also suggest enhanced glycolysis [70, 71, 72]. Of interest, glycolysis inhibition reduces tubular apoptosis but not its dedifferentiation, suggesting divergent effects [19]. Therefore, although this metabolic switch is initially cytoprotective, allowing PTECs to maintain energy production, its persistence may be maladaptive and is associated with worsen kidney prognosis. However, the exact cause of this metabolic switch during AKI and CKD transition remains unknown. Under low oxygen supply, HIF activation increases the expression of glucose transporters and catabolism genes, while inflammatory signals such as TGF‐β and IL‐β can contribute to the perpetuation of this metabolic perturbation by modulating co‐regulators of FAO and glycolysis such as hepatocyte nuclear factor 4 alpha (HNF4α) or ESRRA [73].

Glutamine supplies substrates to the TCA cycle via anaplerotic reactions mostly by producing α‐ketoglutarate (α‐KG). These reactions also generate ammonia, contributing to acid/base balance [74]. While glutamine is glutamate's main carbon source in all PT areas, glutamate oxidation in the TCA cycle differs between PTEC segments. Thus, there is a lower contribution of both glutamine and glutamate to the oxidative TCA cycle in S3 PTECs (Fig. 1C). Under ischemic injury, a reduction of glutamine‐derived oxidative TCA cycle metabolites was observed in FR‐PTs, although glutamine remained the main carbon source for glutamate in these cells (Fig. 2) [69]. Interestingly, even healthy PTECs within murine ischemic injury kidneys displayed this abnormal glutamate to TCA metabolism partitioning, both in the cortex and in the outer medulla, highlighting the relevance of TCA metabolite homeostasis to chronic kidney injury and repair.

Chronic kidney disease has a profound impact on further metabolic routes [75]. Purine and polyamine metabolism is also altered in CKD. Thus, purine catabolism by‐products xanthine and uric acid are reduced both in human and mouse renal fibrotic tissue [76]. In turn, a higher level of serum uric acid, called hyperuricemia, is a risk factor of CKD [77]. Polyamines, essential for cell proliferation, are reduced in injured human PTECs. TGF‐β1 induces the degradation of ornithine decarboxylase, involved in polyamine biosynthesis, by targeting the Azin‐1/Antizyme1 pathway. Low levels of polyamines in turn activate and sustain the TGF‐β1‐induced profibrotic effects in an amplification loop [78]. The urea cycle, the primary metabolic pathway involved in the removal of ammonia (derived from amino acid breakdown) by its transformation into urea, is also impaired [79]. Conversely, several genes of the pentose phosphate pathway, which produces NADPH as reducing equivalents and pentoses as essential parts of nucleotides, showed increased expression in murine PTECs *in vivo* after renal ischemia [80].

## Future perspectives

In the last decade, the significance of metabolism in nephrology has gained widespread interest, resulting in a greater understanding of pathways with a role in renal disease. Bulk tissue metabolomic and transcriptomic platforms along with functional *in vitro* assays have allowed researchers to characterize the metabolic profile of CKD [38, 81]. Over the last few years, single‐cell/nuclei RNA sequencing has revolutionized the identification of cell types and states across kidney disease [4, 72]. Further, spatially resolved high‐resolution transcriptomic platforms along with improved deconvolution software pipelines continue to develop rapidly to provide a spatial cellular context within a tissue [82]. However, this technology does not provide a functional metabolic phenotype. While new approaches are emerging to decipher single‐cell metabolomics [83, 84], the activity of metabolic pathways cannot be determined only by measuring metabolite abundance. Recently, Wang *et al*. [69] followed a different strategy, performing ^13^C‐labeling fluxomic experiments *ex vivo* on cultured tissue slices, in combination with verified marker proteins and membrane lipid signatures, approaching a single‐cell (5 μm pixel size) resolution. This opens the door to measure metabolism with exceptional sensitivity and resolution by applying this technology to *in vivo* systematic analysis based on atom‐level metabolic network models, which have long been utilized for metabolic flux measurement in bulk material. All these observations suggest that a cell‐type‐specific metabolic shift could be a distinctive feature of new cell subpopulations with a differential role in adaptive or fibrotic kidney regeneration. Renal epithelial cells display shared and unique cell states and injury and repair responses across nephron segments in different CKD models [32]. In addition to their role in molecule production, metabolites are now known to regulate gene activity through DNA methylation and post‐translational histone changes [85]. Future research should focus on better analyzing mitochondrial malfunction and organelle interaction in disease since mitochondrial metabolism is a central hub in PTEC function and adaptation. Thus, development of noninvasive methods to monitor mitochondrial function in patients would also be useful. By contrast, the metabolic phenotype of other cell types involved in kidney injury and repair, such as myofibroblast and inflammatory cells, has been less studied. Cell‐type‐specific knockout models would help to clarify divergent effects derived from pharmacological metabolic inhibitors. Further, the impact of this metabolic reprogramming on surrounding cells, as well as the consequences of metabolite accumulation, is unknown. Since kidney fibrosis impacts nearly all renal cell types encompassing epithelia, stroma, endothelia and the immune system, it is desirable to construct a comprehensive network of cell–cell communications for translational studies. To explore this new avenue, more complex systems are needed, including integrated high‐resolution multiomics (metabolomic, transcriptomic, and fluxomic) analysis with spatial and temporal components, which will definitely unleash their full capacity as therapeutic targets.

From a translational perspective, this new knowledge offers the potential of metabolism‐related drugs, which have been more deeply explored in the cancer field, to develop CKD therapeutic strategies (Fig. 1B) [86]. Further preclinical and, at some point, clinical studies are needed to target energy metabolism in fibrosis. Furthermore, determination of circulating metabolites as an innovative noninvasive methodology to identify and monitor the diagnosis, prognosis, and therapeutic response of CKD is also an impending application, albeit no definitive data have yet been moved to the clinic. Understanding the cell specificity of metabolite production in the kidney is essential to contextualize whole‐tissue metabolic changes, target validation and to improve the rational selection of biomarkers [87]. Therefore, elucidation of the relationship between metabolism and fibrosis will provide a new perspective on the identification of new cell subpopulations, uncover mechanisms of fibrogenesis, and open the possibility of exploring metabolism‐based effective therapeutic strategies.

## Conflict of interest

The authors declare no conflict of interest.
